# Aggravated Cardiac Remodeling post Aortocaval Fistula in Unilateral Nephrectomized Rats

**DOI:** 10.1371/journal.pone.0134579

**Published:** 2015-08-07

**Authors:** Jie Wu, Zhong Cheng, Ye Gu, Wusong Zou, Mingjing Zhang, Pengfei Zhu, Shao Hu

**Affiliations:** Heart Center at Puai Hospital, Wuhan Puai Hospital, Huazhong University of Science and Technology, Wuhan, Hubei, China; University Medical Center Utrecht, NETHERLANDS

## Abstract

**Background:**

Aortocaval fistula (AV) in rat is a unique model of volume-overload congestive heart failure and cardiac hypertrophy. Living donor kidney transplantation is regarded as beneficial to allograft recipients and not particularly detrimental to the donors. Impact of AV on animals with mild renal dysfunction is not fully understood. In this study, we explored the effects of AV in unilateral nephrectomized (UNX) rats.

**Methods:**

Adult male Sprague-Dawley (SD) rats were divided into Sham (n = 10), UNX (right kidney remove, n = 10), AV (AV established between the levels of renal arteries and iliac bifurcation, n = 18) and UNX+AV (AV at one week after UNX, n = 22), respectively. Renal outcome was measured by glomerular filtration rate, effective renal plasma flow, fractional excretion of sodium, albuminuria, plasma creatinine, and cystatin C. Focal glomerulosclerosis (FGS) incidence was evaluated by renal histology. Cardiac function was measured by echocardiography and hemodynamic measurements.

**Results:**

UNX alone induced compensatory left kidney enlargement, increased plasma creatinine and cystatin C levels, and slightly reduced glomerular filtration rate and increased FGS. AV induced significant cardiac enlargement and hypertrophy and reduced cardiac function and increased FGS, these changes were aggravated in UNX+AV rats.

**Conclusions:**

Although UNX only induces minor renal dysfunction, additional chronic volume overload placement during the adaptation phase of the remaining kidney is associated with aggravated cardiac dysfunction and remodeling in UNX rats, suggesting special medical care is required for UNX or congenital monokidney subjects in case of chronic volume overload as in the case of pregnancy and hyperthyroidism to prevent further adverse cardiorenal events in these individuals.

## Introduction

Clinical and experimental studies evaluating the cardiac-renal interaction are of importance in health care research. Renal dysfunction is common in patients with heart failure and often associated with high morbidity and mortality, moreover, cardiac and renal dysfunction may worsen each other[1, 2].Clinically, heart failure may also occur in patients with nearly normal or mildly impaired renal dysfunction, as in the case of living donor kidney transplantation[3]. Although living donor kidney transplantation is regarded as beneficial to allograft recipients and not particularly detrimental to the donors and mortality of kidney donors is very low (<0.1%)[4, 5], unilateral nephrectomy might cause an early abrupt decrease in plasma arginine and simultaneous reduction in glomerular filtration rate in living kidney donors[3]. Up to two-thirds of donors after nephrectomy fulfil the criteria for chronic kidney disease stage 3 (eGFR, 30–59 mL/min per 1.73 m^2^) depending on baseline age and renal function[6]. Previous study showed that there was a graded inverse relationship between cardiovascular risk and eGFR, deleterious cardiovascular effects were clearly evident once eGFR falls to <60 mL/min per 1.73 m^2^[7]. Thus, kidney donors might face increased risk in case of additional cardiac insults. The renal and cardiac consequences and heart-kidney interaction with ischemic cardiac insult in unilateral nephrectomized rats were reported previously[8, 9], while the impact of chronic volume-overload in unilateral nephrectomized rats remained largely unknown. In this study, we observed the heart-kidney interaction in a rat aortocaval fistula (AV) model established at one week post right kidney remove (UNX).

## Materials and Methods

### Experimental Animals and Study Groups

Experiments were approved by the Tongji Medical College Council on the Animal Care Committee of Huazhong University of Science and Technology (Wuhan, China). Animals were maintained in accordance with the Guide for the Care and Use of Laboratory Animals published by the US National Institute of Health (NIH Publication No.85-23, revised 1996). All surgery was performed under sodium pentobarbital anesthesia, and all efforts were made to minimize suffering. Male Sprague-Dawley (SD) rats (weighing 200 to 250 g) were housed under standard conditions with free access to food and drinking water. Rats received a normal salt diet (0.3% NaCl) throughout the study. Rats were randomly divided into Sham (n = 10), UNX (n = 10), AV (AV established between the levels of renal arteries and iliac bifurcation, n = 18) and UNX+AV (AV established at one week after UNX, n = 22), respectively. The following steps were taken to minimize the suffering of the rats. First, the rats were handled gently to reduce their discomfort and distress. Second, anesthesia was administered prior to blood sample collection, echocardiography, invasive hemodynamic measurements (40 mg/kg sodium pentobarbital intraperitoneally) and before animal sacrifice(70 mg/kg sodium pentobarbital intraperitoneally). Additionally, anesthesia, examinations and animal sacrifice were undertaken in separate rooms to avoid instilling fear in other rats. The rats were monitored three times a day during the eight-week study period. Moribund animals were euthanized under deep anesthesia. The signs and symptoms of moribund were as follows: (a) impaired ambulation (unable to reach either food or water easily); (b) any obvious severe illness, including signs and symptoms such as lethargy (drowsiness, aversion to activity, a lack of physical or mental alertness), anorexia (loss of appetite, particularly prolonged behavior), bleeding, difficulty breathing, or chronic diarrhea; (c) an inability to remain upright; (d) either rapid weight loss or a net weight loss of more than 20% of body weight; and (e) unconsciousness or unresponsiveness to external stimuli. After eight weeks, survived rats were placed in individual metabolic cages. After 5 days of adaptation, two consecutive 24-hour urine was collected from each rat. Echocardiography was performed two days after the metabolic cage studies. After echocardiography examination, rats received invasive hemodynamic and renal function measurements. Blood sample was obtained from the vena cava post above measurements. Finally, all rats were killed under deep anesthesia (70 mg/kg sodium pentobarbital intraperitoneally), and organs were removed, weighed, and processed for histological quantification.

### Surgical Procedures

Laparotomy was performed under anesthesia with 1% pentobarbital sodium salt (40mg/kg, intraperitoneal injection). The right kidney was carefully separated from the adrenal gland and the surrounding tissue. The right renal artery and vein, as well as the urethra, were ligated with a 4.0 silk suture, followed by removal of the right kidney. These rats were allowed to recover from surgery in a warmed cage for 1 to 2 hours. One week after nephrectomy, rats assigned to UNX+AV group were re-anesthetized with 1% pentobarbital sodium salt (40mg/kg, intraperitoneal injection) and the aortocaval fistula was produced according to the method described by Garcia and Diebold [10] with some modifications. Briefly, ventral abdominal laparotomy was performed in anesthetized rats, the intestines were displaced laterally and wrapped with normal saline-soaked sterilized gauze to retain moisture. The aorta and vena cava between the levels of renal arteries and iliac bifurcation were then exposed by blunt dissection of the overlaying adventitia. Both vessels were temporarily occluded proximal and distal to the intended puncture site, and a 18-gauge needle held on a plastic syringe was inserted into the exposed abdominal aorta and advanced through the medial wall into the vena cava to create the shunt. The needle was inserted and withdrawn across the medial wall several times through the same hole, to ensure the size and presence of the fistula, before it was finally withdrawn from the aorta. The ventral aortic puncture site was immediately sealed with a drop of cyanoacrylate (Medical Adhesive Glue; Baiyun Medical Adhesive Co., China) after withdrawal of the needle. Creation of a successful fistula was confirmed by visualizing the pulsatile flow of oxygenated blood into the vena cava from the abdominal aorta. The intestines were repositioned, and the abdominal musculature and skin incisions were closed by standard techniques with absorbable suture and autoclips. Sham rats underwent similar surgical procedures as rats in UNX+AV group without right kidney removal and AV creation.

### Echocardiography Examination

Echocardiography examination was performed by an investigator blinded to the study protocol. Left parasternal and left apical echocardiographic images of light anesthetized (1% pentobarbital sodium salt, 40mg/kg, intraperitoneal injection) rats lying in a supine position were obtained with an echocardiographic system (GE Vivid 7) equipped with a 11.4 MHz transducer. A two-dimensional short-axis view of the left ventricle was obtained at the level of the papillary muscles. Left ventricular M-mode tracings were used to define the internal systolic and diastolic diameters. Fractional shortening (FS) is defined as [(LVEDd-LVEDs)/LVEDd]*100%, in which LVEDd is the left ventricular end-diastolic diameter and LVEDs is the corresponding left ventricular end-systolic diameter. Left ventricular ejection fraction (LVEF) is defined as [(LVEDV-LVESV)/LVEDV]*100%, in which LVEDV is the left ventricular end-diastolic volume and LVESV is the left ventricular end-systolic volume. Measurements represent the mean of at least three consecutive cardiac cycles[11].

### Hemodynamic measurements

Forty-eight hours after echocardiography examination, the rats underwent left and right heart catheterization under pentobarbital sodium salt anesthesia (40mg/kg, intraperitoneal injection). The right carotid artery and right jugular vein were exposed, cannulated, saline-filled PE-50 tubings (0.58 mm ID, 0.96 mm OD) connected to a pressure transducer (BL-420F biological function experimental system, Chengdu Technology & Market Co. Ltd., Chengdu, China) were inserted into the right carotid artery and right jugular vein, respectively. The jugular vein catheter was advanced into the right ventricle and the right atrium for the recording of right ventricular systolic pressure (RVSP), right ventricular end-diastolic pressure (RVEDP), and right atrium pressure (RAP), and the maximum rate of rise (dP/dtmax) and decrease (dP/dtmin) of RV systolic pressure. The carotid catheter was advanced into the left ventricle for the recording of left ventricular systolic pressure (LVSP), left ventricular end-diastolic pressure (LVEDP), the maximum rate of rise (dP/dtmax) and decrease (dP/dtmin) of LV systolic pressure and then was withdrawn into the aortic root to measure the heart rate (HR), systolic aortic pressure (SAP) and diastolic aortic pressure (DAP), and the mean arterial pressure (MAP) was calculated as: [(2*DAP)+SAP]/3.

### Renal function measurements

The jugular vein catheter was connected to a syringe and each rat was given an i.v. bolus (2ml/kg) of the inulin/ para-aminohippurate(PAH) solution, followed by an i.v. infusion (25μl/min) of the inulin/PAH solution via an infusion pump. The inulin/PAH solution contained 3% inulin and 2% PAH in saline. An additional infusion of 25μl/min of saline into the jugular vein catheter was also performed and was continued for the duration of the experiment. Following the l hour equilibration period, hemodynamics and renal function were determined for 90 min (three 30 min clearance periods). Urine was collected in pretared vials at 30 min intervals through the bladder catheter. Arterial blood samples (500μl) were drawn through the carotid artery catheter at the midpoint of each urine collection period into prechilled heparinized tubes. A small blood sample was taken into microhematocrit tubes for measurement of hematocrit. The urine samples were measured gravimetrically for urine volume. Urine samples were then refrigerated until they were assayed. Plasma samples were separated by centrifugation and kept at -80℃ freezer until analysis.

### Histology

After blood sampling from vena cava, the rats were sacrificed under additional deep anesthesia (70 mg/kg sodium pentobarbital intraperitoneally). Rats were weighed, hearts from five rats of each group underwent gross morphology study. The hearts were taken out, washed, and fixed in formalin buffer for 1 week. The hearts were then blotted dry and sagitally cut to show the size of the cavity and thickness of the LV and RV walls as well as that of the septum. Hearts from the other rats were removed and immediately placed in ice-cold saline to wash out the blood. Total heart, right ventricular, left ventricular weight including the septum were measured. Then all tissues were embedded in paraffin, and cut into 4 μm slices, which were stained with HE and Masson and then images were captured with a Leica microscope. Cardiomyocyte size was determined by measuring the narrowest diameter of 75 cardiomyocytes from left and right ventricle. Kidney, liver and lung were also removed from all animals and immediately placed in ice-cold saline to wash out the blood, wet weights were measured.

Kidneys were weighed and then fixed by immersion for 48 hours in 4% neutral formaldehyde after longitudinal bisection. Subsequently, they were processed for paraffin embedding according to standard procedures. Sections of 3 μm were stained with periodic acid–Schiff (PAS) and microscopically evaluated by determining the incidence of focal glomerulosclerosis (FGS) as previously descried[12]. For each animal, 100 glomeruli were examined in the inner and outer cortical region and the number of sclerotic glomeruli was counted. Criteria on which glomeruli were designated as sclerotic consisted of adhesion of the glomerulus to Bowman’s capsule, thickening of Bowman’s capsule, the presence of increased amounts of PAS-positive material in the mesangial region, and/or folding of the glomerular basement membrane with entrapment of amorphous material. Glomerulosclerosis was scored by quadrants, on a scale of zero to four, where zero means no quadrant affected and four means that the whole glomerulus was affected. Gross glomerulosclerosis score was calculated as a summation of (n glomeruli)*(score)/100. An examiner blinded for the groups evaluated all sections.

### Biochemistry Measurements

24-hour urine albumin was measured by using a sensitive enzyme-linked immunosorbent assay (ELISA) kit (Abcam, America). Plasma creatinine was measured with the Jaffé method with deproteinization. Plasma rBNP-45 concentrations were measured by using a sensitive enzyme-linked immunosorbent assay (ELISA) kit (Assaypro, America). Plasma cystatin C concentrations were measured by using a sensitive enzyme-linked immunosorbent assay (ELISA) kit (R&D Systems, America). An enzymatic method was used for the determination of inulin, and a calorimetric method was used for determination of PAH in the plasma and urine samples. GFR was estimated as the clearance of inulin (urine volume*urine inulin/plasma inulin). Effective renal plasma flow (ERPF) was estimated as the clearance of PAH (urine volume*urine PAH/plasma PAH). Renal blood flow was calculated as ERPF/(1-hematocrit). Renal vascular resistance was calculated as mean arterial pressure divided by the renal blood flow. Fractional excretion of sodium was calculated as the clearance of sodium divided by the GFR.

### Statistical Analyses

All data are presented as mean±SD. Differences between groups in mean values with normal distribution were compared by 2-way ANOVA followed by Tukey test, otherwise a kruskal-wallis test followed by Mann-Whitney U test with Bonferroni correction was used. P<0.05 was considered statistically significant.

## Results

### Survival and general characteristics

One moribund rat was euthanized under deep anesthesia(70 mg/kg sodium pentobarbital intraperitoneally) on the first post-operation day in UNX+AV group due to difficulty breathing and drowsiness, post mortem examination revealed massive bleeding around the puncture site, and the symptoms might be associated with failure of sealing the puncture site by cyanoacrylate. Another rat in AV group was found dead on the morning of the 18^th^ day post operation day and post mortem examination revealed hypertrophied heart, congested liver and lung, presence of ascites and pleural effusion, as well as edema of the limbs, suggesting that overt congestive heart failure might be the cause of death in this rat. Seven rats (4 in AV group and 3 in UNX+AV group) were excluded from the final analysis because the lack of mixing of venous and arterial blood in the vena cava visualized before sacrifice. Finally, data from 10 rats in Sham and UNX group, 13 rats in AV group and 19 rats in UNX+AV group were analyzed. Ascites and pleural effusion were not evidenced on the rats survived to study end.

### Body weight and organ weights, morphological changes

Body weight (BW) and organ weights are shown in Table 1. Eight weeks post various procedures, body weight was similar among groups, heart weight and heart/BW ratio, LVW(mg), LVW/BW(mg/g), RVW(mg) and RVW/BW(mg/g) were similar between UNX and Sham groups, while significantly increased in AV and UNX+AV groups compared both Sham and UNX groups. These values (except RVW/BW) also tended to be higher in UNX+AV group compared to AV group. Wet lung weight was significantly higher in UNX+AV groups compared to Sham and UNX groups, while wet lung/BW ratio significantly increased in AV and UNX+AV groups compared to Sham and UNX groups. Liver wet weight and liver wet weight/BW ratio were similar among groups. Left kidney weight was significantly higher in UNX and UNX+AV groups compared to Sham and AV groups.

**Table 1:** Body weight and organ weights.

	SHAM(n = 5)	UNX(n = 5)	AV(n = 7)	UNX +AV(n = 13)
**BW(g)**	600±31	595±67	569±20	616±59
**HW(mg)**	1613±156	1519±110	2545±365^*^ ^†^	2823±380^*^ ^†^
**HW/BW(mg/g)**	2.68±0.20	2.56±0.17	4.47±0.63^*^ ^†^	4.62±0.71^*^ ^†^
**LVW(mg)**	1110±58	1142±104	1779±232^*^	1928±320^*^ ^†^
**LVW/BW(mg/g)**	1.85±0.01	1.93±0.11	3.13±0.41^*^ ^†^	3.14±0.48^*^ ^†^
**RVW(mg)**	328±55	262±10	465±78^*^ ^†^	496±65^*^ ^†^
**RVW/BW(mg/g)**	0.55±0.08	0.45±0.07	0.82±0.13^*^ ^†^	0.81±0.10^*^ ^†^
**Lung Wt(mg)**	2229±147	2131±270	2466±351	2545±289^*^ ^†^
**Lung Wt/BW(mg/g)**	3.71±0.13	3.34±0.44	4.34±0.66^*^ ^†^	4.08±0.54^†^
**Liver Wt(mg)**	18152±1591	18452±1988	16508±1105	16874±2056
**Liver Wt/BW(mg/g)**	30.45±2.03	28.96±3.81	29.02±1.86	26.99±3.32
**Left kidney Wt(mg)**	1744±170	2507±811	1786±213	2596±473^*^ ^‡^
**Left kidney Wt/BW(mg/g)**	2.91±0.33	4.31±1.63	3.14±0.39	4.10±0.65^*^ ^‡^

Values are mean±SD. UNX, unilateral nephrectomy; AV, aortocaval fistula; BW, Body weight; HW, Heart weight; LVW, left ventricular weight; RVW, right ventricular weight; Wt, wet weight.

*p<0.05 vs. Sham

†p<0.05 vs. UNX

‡ p<0.05 vs. AV.

**Fig 1:**
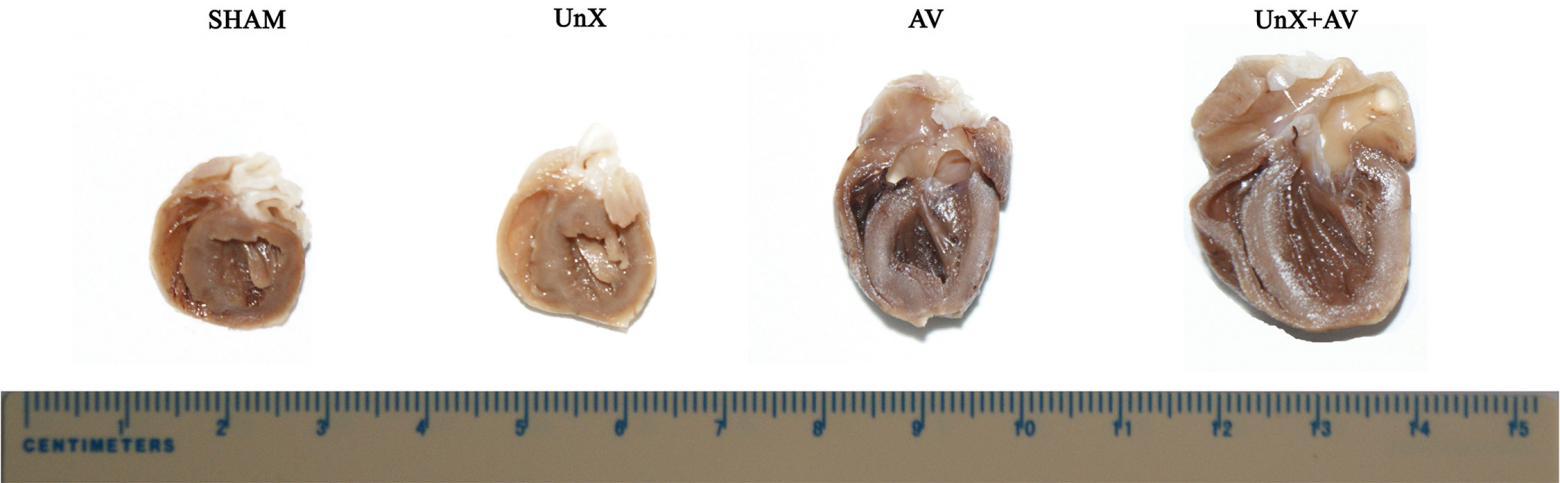
Gross heart morphology showing ventricular enlargement and cardiac hypertrophy (of rat hearts) in the AV group, especially (of rat hearts) in the UNX+AV group.

The gross morphological changes of the hearts from various groups are shown in Fig 1. As expected, heart enlargement and LV and RV cavity dilation as well as and hypertrophied wall thickness were observed in the vertically cut hearts of the AV group and these changes were more apparent in UNX+AV group while there were no obvious changes in the heart of UNX and Sham groups.

### Echocardiography measurements

**Fig 2:**
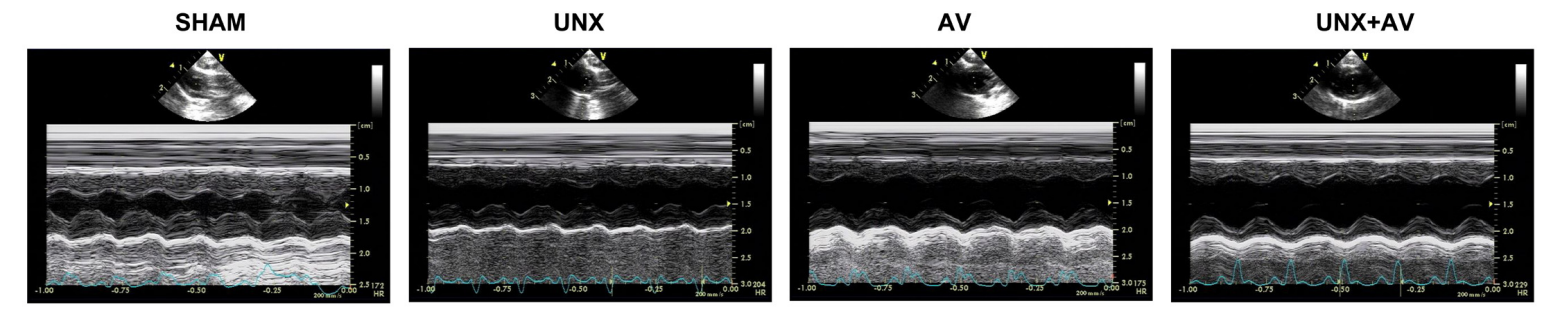
Transthoracic echocardiography. Representative B- and M-mode images of the rats from SHAM, UNX, AV and UNX+AV groups.

As shown in Fig 2 and Table 2, LVEDD and LVESD were significantly higher while LVEF and LVFS values were significantly lower in the AV and UNX+AV groups than in Sham and UNX groups. There is a trend of severer LV remodeling and dysfunction in UNX+AV group compared to AV group.

**Table 2:** Echocardiographic Parameters.

	SHAM (n = 8)	UNX (n = 9)	AV (n = 12)	UNX +AV (n = 16)
**LVEDD(mm)**	6.378±0.611	5.960±0.659	8.650±0.454^†^	10.075±0.602^*^ ^†^
**LVESD(mm)**	3.139±0.888	3.162±0.686	4.740±0.724^*^ ^†^	5.748±0.596^*^ ^†^ ^‡^
**EF(%)**	91.50±5.37	86.44±4.22	77.58±3.09^*^	71.63±2.87^*^ ^†^
**FS(%)**	59.63±9.49	50.78±5.74	41.83±2.82^*^	36.50±2.19^*^ ^†^

Values are mean±SD. LVEDD, left ventricular end-diastolic dimension; LVESD, left ventricular end-systolic dimension; EF, ejection fraction; FS, fractional shortening.

*p<0.05 vs. Sham

†p<0.05 vs. UNX

‡ p<0.05 vs. AV.

### Hemodynamic measurements

**Fig 3:**
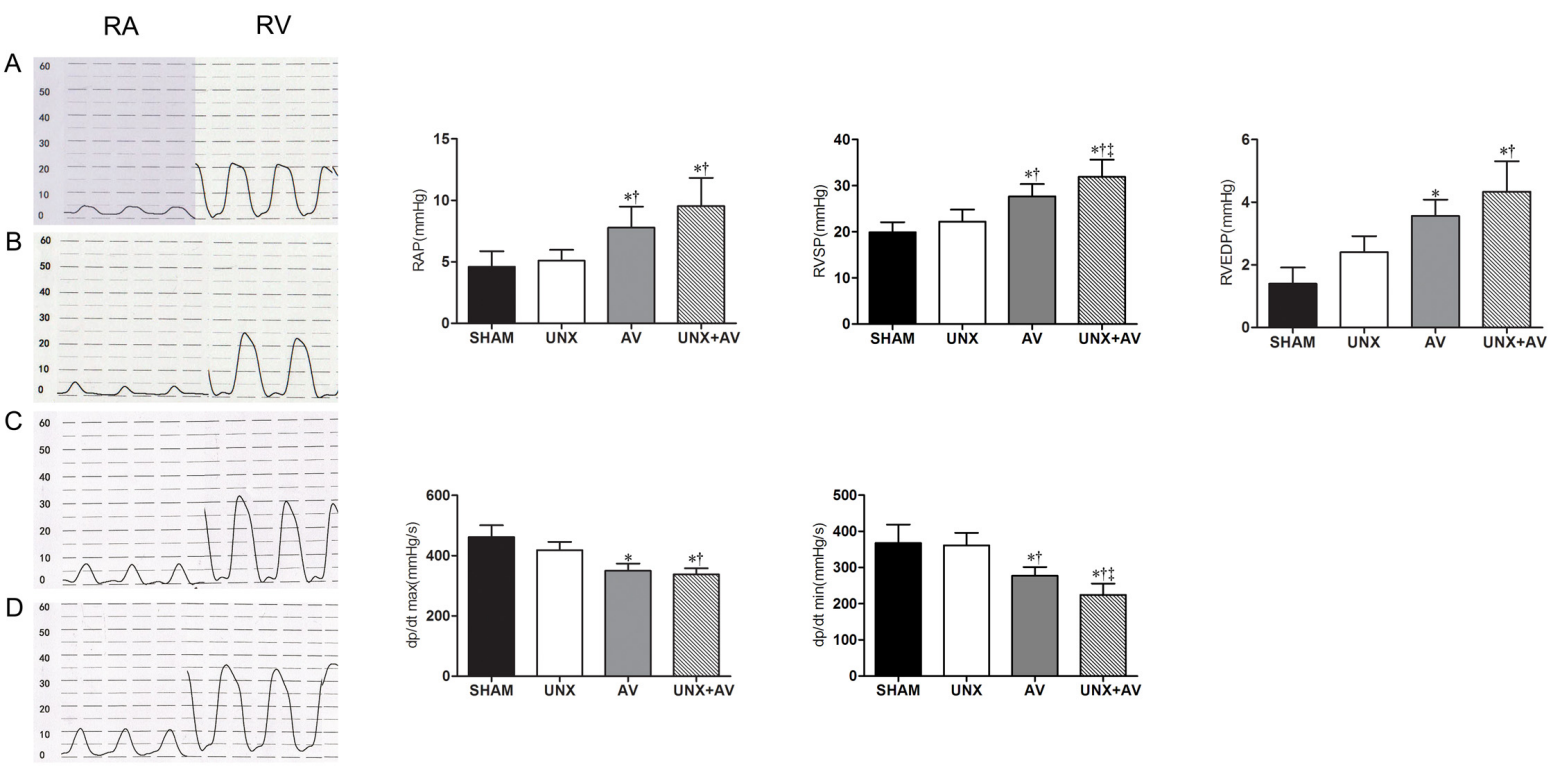
Right heart hemodynamic analysis. [A, Sham (n = 10); B, UNX (n = 10); C, AV (n = 9); D, UNX+AV (n = 15)]. Values are mean±SD. RA, right atrium; RV, right ventricle; RAP, right atrial pressure; RVSP, right ventricular systolic pressure; RVEDP, right ventricular diastolic pressure; dP/dtmax, first derivative of pressure rise; dP/dtmin, first derivative of pressure decrease. *p<0.05 vs. Sham; †p<0.05 vs. UNX; ‡ p<0.05 vs. AV.

**Fig 4:**
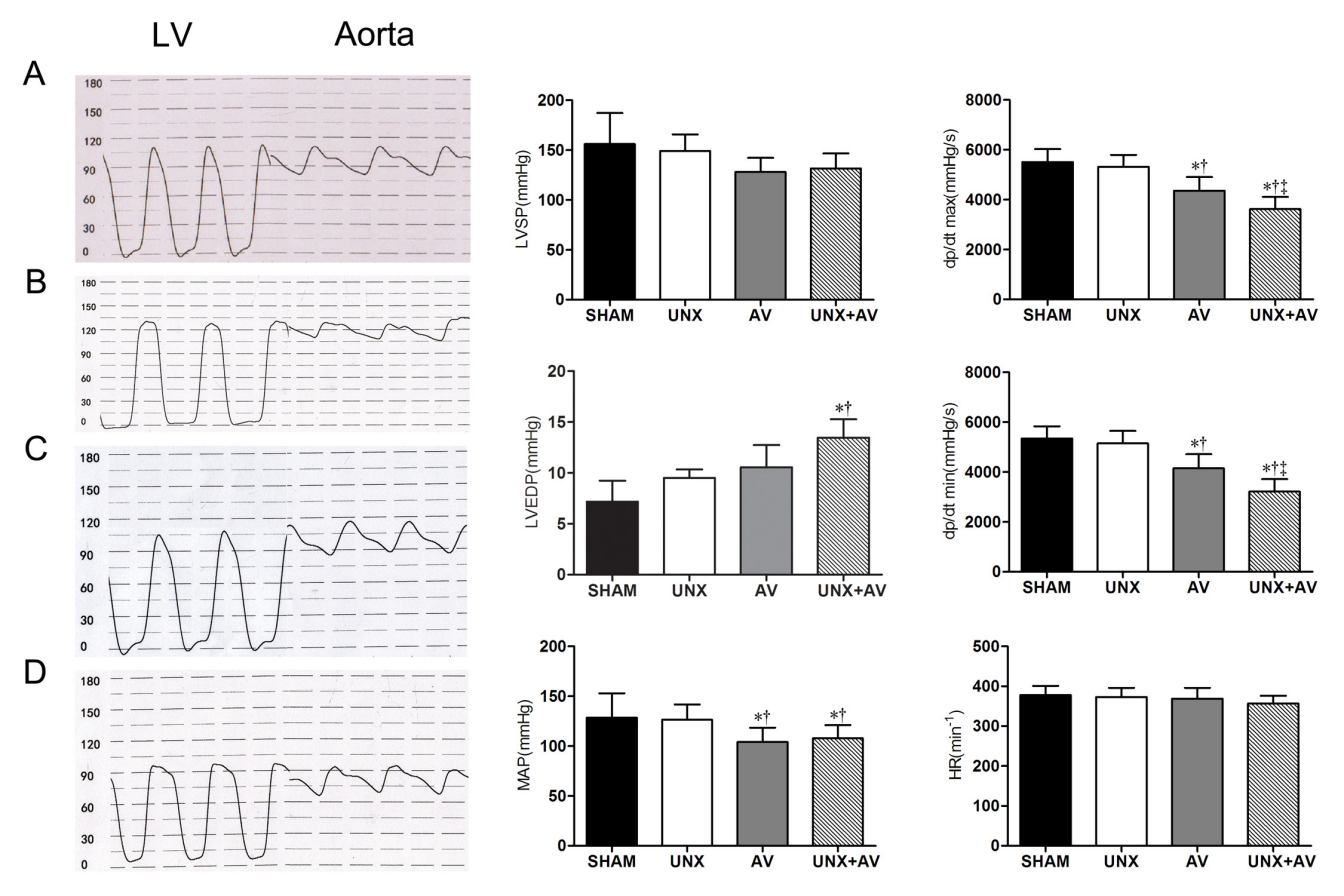
Left heart hemodynamic analysis. [A, Sham (n = 10); B, UNX (n = 10); C, AV(n = 9); D, UNX+AV (n = 15)]. Values are mean±SD. LV, left ventricle; Aorta, Aorta artery; LVSP, left ventricular systolic pressure; LVEDP, left ventricular diastolic pressure; MAP, mean arterial pressure; dP/dtmax, first derivative of pressure rise; dP/dtmin, first derivative of pressure decrease; HR, heart rate. *p<0.05 vs. Sham; †p<0.05 vs. UNX; ‡ p<0.05 vs. AV.

Hemodynamic measurements results are shown in Figs 3 and 4. As shown in Fig 3, RAP, RVSP and RVEDP were significantly higher while RV dP/dtmax and RV dP/dtmin were lower in AV and UNX+AV groups compared to Sham and UNX groups, these changes tended to be more apparent in UNX+AV group compared to AV group, but did not reach statistical significance.

Fig 4 shows that LVSP and MAP tended to be lower, LVEDP to be higher in AV and UNX+AV groups compared to Sham and UNX groups. LV dp/dtmax and dP/dtmin were significantly lower in UNX+AV group compared to Sham and UNX groups. Heart rate was similar among groups.

### Biochemistry and renal function parameters

Rat brain natriuretic peptide-45 tended to be slightly higher post UNX and AV operations compared to Sham group. Plasma creatinine and cystatin C levels were significantly increased in UNX and UNX+AV groups compared to Sham and AV groups and there was a trend for more apparent changes in UNX+AV group than in UNX group, but these changes did not reach statistical significance (Table 3). GFR tended to be lower in UNX rats and was significantly reduced in UNX+AV rats compared to Sham rats; ERPF and RBF tended to be lower in UNX and UNX+AV groups and tended to be higher in AV group compared to Sham group; RVR was significantly increased in UNX and UNX+AV groups compared to Sham group; FENa was significantly increased in AV and UNX+AV group compared to Sham group; UV tended to be lower in UNX group and higher in AV group compared to Sham group; 24 hours albuminuria tended to be higher in UNX+AV group compared to Sham group (Table 3). Renal function was similar one day before AV placement in UNX+AV group and at 9 weeks post operation in UNX group (data not shown).

**Table 3:** Biochemistry and renal function parameters.

	SHAM (n = 10)	UNX (n = 10)	AV (n = 9)	UNX +AV (n = 15)
**Rat BNP-45 (ng/ml)**	0.14±0.02	0.17±0.03	0.23±0.11	0.25±0.06
**sCr(mg/L)**	4.68±0.87	5.56±0.48^*^	4.43±0.63^†^	6.06±0.75^*^ ^‡^
**Cys-c(**μ**g/ml)**	1.51±0.19	2.66±0.63^*^	1.80±0.47	2.91±0.66^*^ ^‡^
**GFR(ml/min/kg)**	0.079±0.041	0.045±0.015	0.093±0.099	0.028±0.010^*^
**ERPF(ml/min/kg)**	1.672±0.935	0.871±0.712	4.093±3.629^†^	0.799±0.318^‡^
**RBF(ml/min/kg)**	2.944±1.727	1.468±1.200	6.792±5.795^†^	1.326±0.512^‡^
**RVR(mmHg/ ml/min/kg)**	45.275±24.851	89.681±35.561^*^	32.853±29.827^†^	74.259±26.763^‡^
**FENa**	0.087±0.055	0.120±0.075	0.535±0.675^*^	0.263±0.107^*^
**UV(**μ**l/min)**	12.670±9.460	6.013±2.151	29.209±33.573^†^	11.339±3.485
**24-h Albumiuria (**μ**g)**	155.49±115.25	113.35±127.44	167.52±100.34	291.01±183.13

Values are mean±SD. BNP-45, rat brain natriuretic peptide-45; sCr, plasma creatinine; Cys-c, Cystatin C; GFR, glomerular filtration rate; ERPF, effective renal plasma flow; RBF, renal blood flow; RVR, renal vascular resistance; FENa, fractional excretion of sodium; UV, urine volume

*p<0.05 vs. Sham

†p<0.05 vs. UNX

‡ p<0.05 vs. AV.

### Renal and cardiac histology

**Fig 5:**
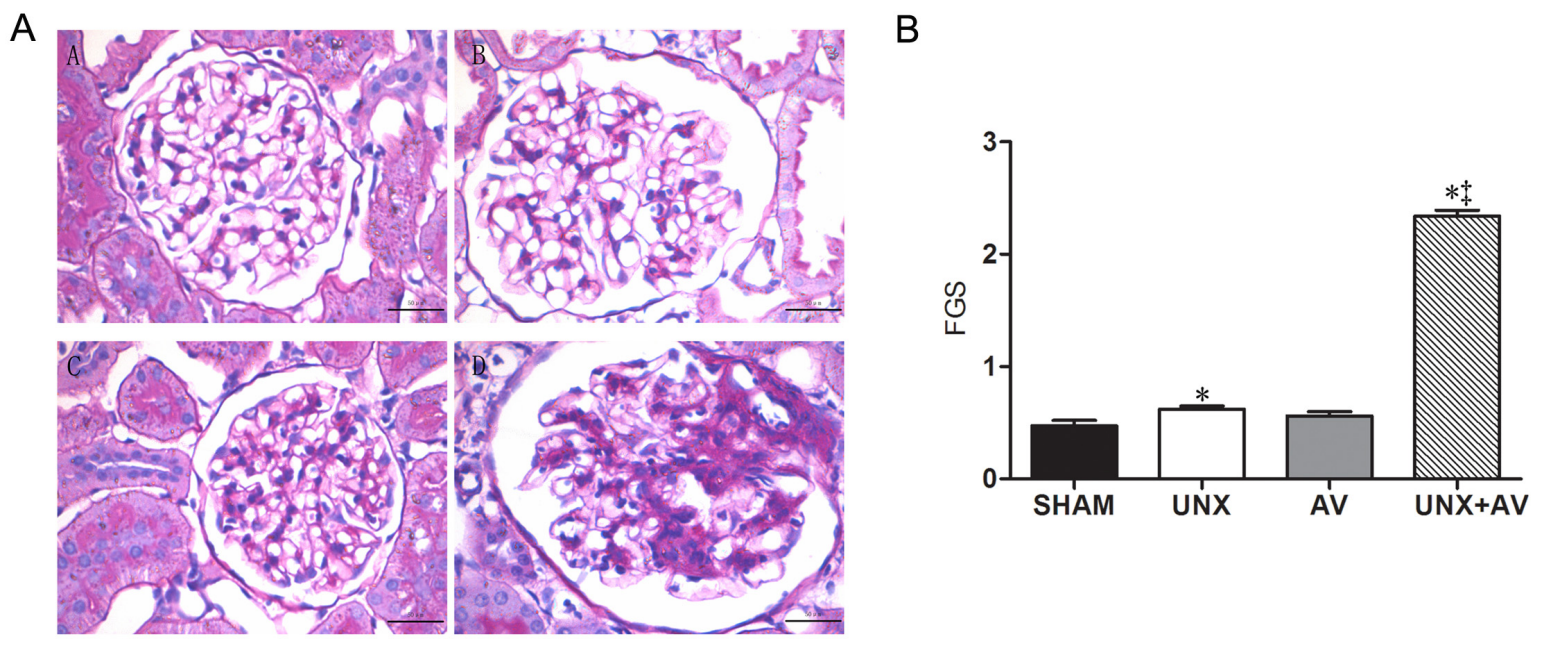
Histological features of renal tissue. [Sham (n = 10); UNX (n = 10); AV (n = 12); UNX+AV (n = 18)]. A. Periodic Acid-Schiff staining (x400) of rat with the highest proteinuria from every experimental group. B. FGS quantification. *p<0.05 vs. Sham;†p<0.05 vs. UNX;‡p<0.05 vs. AV.

Fig 5A showed that the glomerular capillary loops were thin and delicate, endothelial and mesangial cells were normal in Sham group and AV group. In UNX group, there were signs of compensatory glomerular enlargement and the mild mesangial region hyperplasia. In UNX+AV group, there was an area of collagenous sclerosis running across the middle of this glomerulus implying a focal segmental glomerulosclerosis. Focal segmental glomerulosclerosis quantification showed FGS was significantly increased in both UNX and AV groups and strikingly increased in UNX+AV group (Fig 5B).

**Fig 6:**
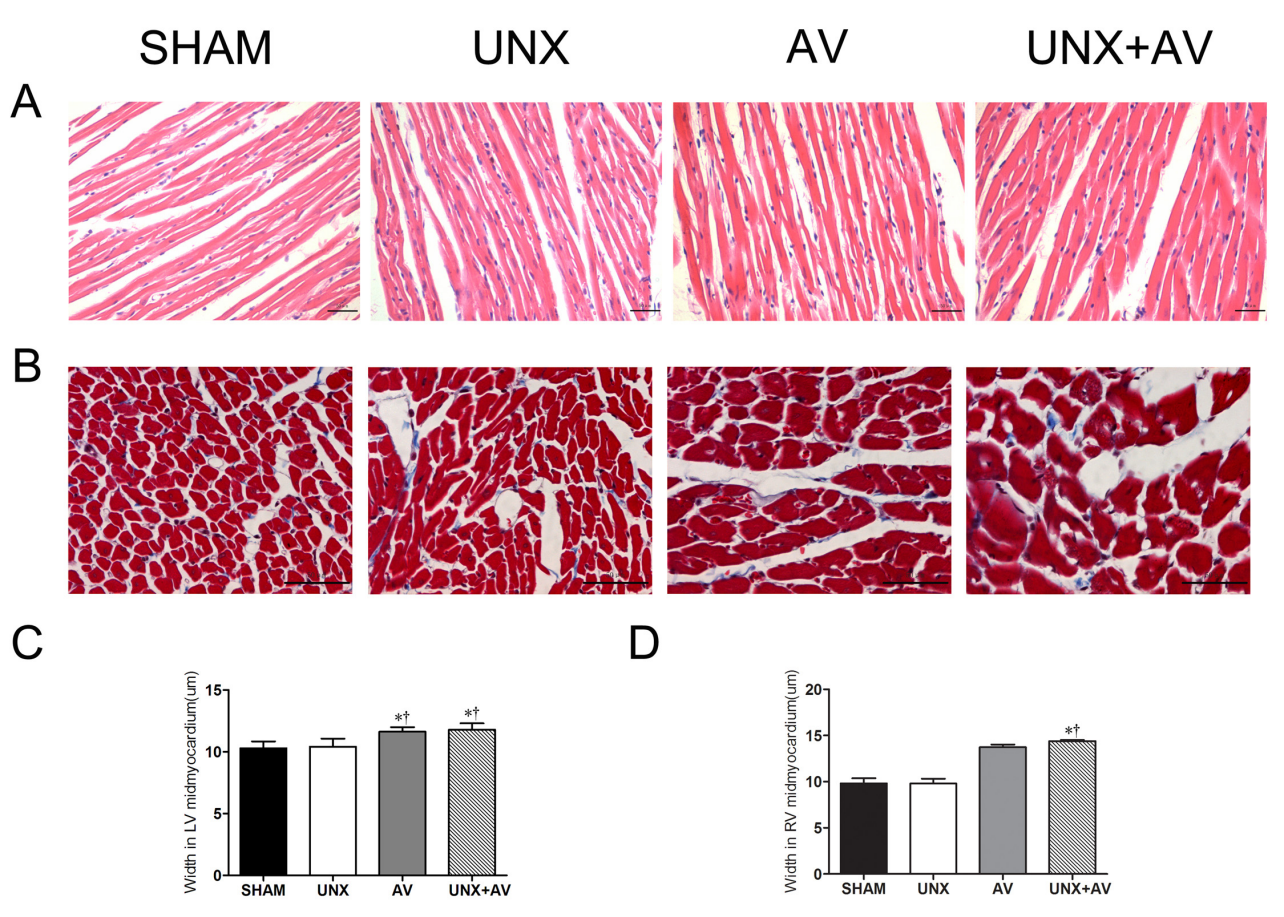
Histological features of cardiac tissue. [Sham (n = 10); UNX (n = 10); AV (n = 13); UNX+AV (n = 19)].A. Hematoxylin and eosin (HE) staining, x200. B. Masson’s trichrome staining, x400. C. Bar graphs of left ventricular cardiomyocyte width. D. Bar graphs of right ventricular cardiomyocyte width. *p<0.05 vs. Sham;†p<0.05 vs. UNX;‡p<0.05 vs. AV.

Fig 6A presented HE-stained myocardial sections showing cardiomyocyte hypertrophy in the AV group and UNX+AV group, Fig 6B presented Masson-stained myocardial section and collagen content was similar among groups. Fig 6C showed that cardiomyocyte width from left ventricle and Fig 6D showed that from right ventricle were significantly increased in AV and UNX+AV groups compared to Sham and UNX groups.

## Discussion

The major finding of present study is that chronic volume overload induced cardiac remodeling is significantly aggravated, which is joined by a trend of more apparent renal and cardiac dysfunction, in the presence of minor renal dysfunction post unilateral nephrectomy.

Most patients with heart failure have mild or moderate renal dysfunction[13].Both mild and severe renal dysfunctions are closely related to poor outcome in heart failure patients[14]and cardiac and renal dysfunction may worsen each other[2]. Previous studies mostly focused the pathogenesis and interactive associations between renal and cardiac dysfunction on disease outcome and therapy goal[15]. Experimental studies already verified the interaction between mild renal dysfunction and ischemic cardiac insult in UNX rats[8, 9], while the impact of chronic volume overload on preexisting mild renal dysfunction remains largely unknown.

Results from present study show that UNX alone is only linked with very mild renal dysfunction, as shown by light focal glomerulosclerosis, compensatory enlargement of the remaining left kidney and slightly but significantly increased plasma creatinine and cystatin C level, and mildly reduced GFR and UV as well as significantly increased RVR. However, chronic volume overload-induced cardiac and renal remodeling tended to be more significant on the basis of mild renal dysfunction post UNX. Thus, mild renal dysfunction is a key determinant for future aggravated cardiac and renal remodeling and function worsening in case of volume overload induced cardiac insult. In a landmark study, Metra and colleagues demonstrated that worsening renal function, characterized by an increase in serum creatinine levels ≥0.3 mg/dL, alone is not an independent determinant of outcomes in patients with heart failure, but it has an additive prognostic value when it occurs in patients with persistent signs of congestion[16]. It is to note that although we evidenced more significant morphological cardiac remodeling in UNX+AV rats than in AV rats, there were mostly just a trend of aggravation on the measured cardiac and renal remodeling and dysfunction parameters in the UNX+AV rats compared to AV rats, the underlying reason might partly explained by the relative short observation period and the limited interval between UNX and AV placement (1 week interval). Future studies with longer observation period and longer interval between UNX and AV placement are warranted to verify if there would be significant differences between UNX+AV and AV groups in rats with only mild renal dysfunction. Taken together, our study provoked a potential detrimental role of slightly increase serum creatinine in case of chronic volume overload in this UNX+AV rat model.

Results from present study are obtained from the rat model of aortocaval fistula, which is a unique model of volume-overload congestive heart failure and cardiac hypertrophy. Creation of aortocaval fistula results in an immediate and sustained decrease in mean arterial pressure together with a substantial increase in venous blood flow to the right heart. These hemodynamic changes could result in compensatory activation of several neuro-hormonal systems as well as adaptive structural alterations in myocardium and vascular system, as shown previously by other investigators[17–19]. Our results showed that UNX resulted in a very mild state of chronic, mild renal function loss. Despite the very mild renal dysfunction observed in this UNX model, additional chronic volume load resulted in greater cardiac and renal remodeling and dysfunction in this model. These results are joined by increased preload of both right and left ventricles as evidenced by higher RVEDP and LVEDP in UNX+AV rats, reduced LV and RV function as shown by reduced EF, FS and LV and RV dP/dtmax and dP/dtmin. Increased RAP also indicated increased right-side volume overload in both AV and UNX+AV rats. Thus, healthy living donors might thus face increased cardiac and renal dysfunction risk in case of future cardiac insult, as in terms of chronic volume load shown in the present study.

Living donor kidney transplantation is regarded as beneficial to allograft recipients and not particularly detrimental to the donors. In 2014, 17105 kidney transplants took place in the U.S. Of these, 11,570 came from deceased donors and 5,535 came from living donors[20].According to our results, healthy living donors might face increased cardiac and renal dysfunction risk in case of future chronic volume overload cardiac insult, as in the case of pregnancy and other high output heart failure situations as in the case of hyperthyroidism[21]and patients with arteriovenous fistula[22]. Ibrahim and colleagues determined the fetal and maternal outcomes in a large cohort of kidney donors and found that post-donation pregnancies (vs. pre-donation) were associated with a lower likelihood of full-term deliveries and a higher likelihood of fetal loss, with a higher risk of gestational diabetes, gestational hypertension, proteinuria and preeclampsia[23].Thus, special medical care and education should be afforded to this population to actively treat and avoid volume overload conditions and related negative renal and cardiac consequences.

## Study Limitations

This study has several limitations that should be acknowledged. First, although the AV is produced by 18-gauge needle in all animals, we are unable to confirm that the flow over the AV-fistula was comparable between AV and UNX+AV groups and between individual animals, future studies with different size of needles are warranted to explore the impact of various fistula flow in this model. Second, the water intake was not measured in this study, so the potential impact of hypovolemic on echocardiographic and hemodynamic measurements remain unknown. Third, one week recovery after UNX might not be enough to reach a new stable phase of renal function. It is possible that increased renal hypoxia and damage during the adaptation-phase might lead to neurohormonal activation and subsequently lead to increased volume retention and observed negative impact on AV-induced renal and cardiac performance. Our finding is thus limited to the AV placement during adaptation of the remaining kidney phase, and future studies with longer UNX and AV placement interval and longer observation period are needed to explore the exact time-frame of events.

In conclusion, although UNX only induces minor renal dysfunction, additional chronic volume overload placement during the adaptation phase of the remaining kidney is associated with aggravated cardiac remodeling and cardiac and renal dysfunction in UNX rats. Special medical care and education should be afforded to living kidney donors and people with congenital monokidney, especially in pregnancy and other high-output situations like in patients with hyperthyroidism and arteriovenous fistula to avoid/correct volume overload conditions and to attenuate the negative consequences of volume overload in this special population.
